# Activation of hTREK-1 by polyunsaturated fatty acids involves direct interaction

**DOI:** 10.1038/s41598-024-66192-w

**Published:** 2024-07-02

**Authors:** Emilie Bechard, Elodie Arel, Jamie Bride, Julien Louradour, Xavier Bussy, Anis Elloumi, Claire Vigor, Pierre Soule, Camille Oger, Jean-Marie Galano, Thierry Durand, Jean-Yves Le Guennec, Hamid Moha-Ou-Maati, Marie Demion

**Affiliations:** 1 PhyMedExp, Université de Montpellier, Inserm U1046, UMR CNRS 9412, CHU Arnaud de Villeneuve, Bâtiment Craste de Paulet, 370 Avenue du Doyen Gaston Giraud, 34290 Montpellier Cedex 05, France; 2 IBMM, Université de Montpellier, UMR CNRS 5247, ENSCM, Montpellier, France; 3 IGF, Université de Montpellier, UMR CNRS 5203, Inserm 1191, Montpellier, France; 4 NanoTemper Technologies GmbH, Munich, Germany; 5 Present Address: INM, Inserm U1298, Montpellier, France

**Keywords:** Ion channels, Lipids

## Abstract

TREK-1 is a mechanosensitive channel activated by polyunsaturated fatty acids (PUFAs). Its activation is supposed to be linked to changes in membrane tension following PUFAs insertion. Here, we compared the effect of 11 fatty acids and ML402 on TREK-1 channel activation using the whole cell and the inside-out configurations of the patch-clamp technique. Firstly, TREK-1 activation by PUFAs is variable and related to the variable constitutive activity of TREK-1. We observed no correlation between TREK-1 activation and acyl chain length or number of double bonds suggesting that the bilayer-couple hypothesis cannot explain by itself the activation of TREK-1 by PUFAs. The membrane fluidity measurement is not modified by PUFAs at 10 µM. The spectral shift analysis in TREK-1-enriched microsomes indicates a K_D,TREK1_ at 44 µM of C22:6 n-3. PUFAs display the same activation and reversible kinetics than the direct activator ML402 and activate TREK-1 in both whole-cell and inside-out configurations of patch-clamp suggesting that the binding site of PUFAs is accessible from both sides of the membrane, as for ML402. Finally, we proposed a two steps mechanism: first, insertion into the membrane, with no fluidity or curvature modifications at 10 µM, and then interaction with TREK-1 channel to open it.

## Introduction

Among the K^+^ channels, members of the two-pore domains K^+^ channels family (K2P) are involved in the repolarization phase of action potential and in the resting membrane potential^1^. TREK-1 channel has been discovered in 1996 by Fink et al. and belongs to the TREK subfamily of mechanosensitive channels with TREK-2 and TRAAK. The gating of TREK-1 channel is poly-modulated by a wide range of physical and chemical stimuli including mechanical stretch, temperature, voltage, pH changes, pharmacological agents and polyunsaturated fatty acids (PUFAs). This channel is widely studied since its activation is involved in neuroprotection^2^, cardioprotection^3^, analgesia^4^ and reduced epilepsy crisis^5^. In the majority of these diseases, the protection afforded by TREK-1 activation is due to the hyperpolarization of the membrane potential^6^.

Among the wide diversity of TREK-1 modulators, PUFAs have been shown to behave as strong activators^7,8^. PUFAs are amphipathic molecules with a hydrophilic carboxyl head and a long hydrophobic chain of carbons and multiple double bonds. The two main classes of PUFAs are n-3-PUFAs and n-6-PUFAs based on the position of the first double bond from the carbon ω starting at the methyl extremity. Numerous studies suggest that n-3-PUFAs, such as docosahexaenoic acid (DHA) and eicosapentaenoic acid (EPA), exert antiarrhythmic properties through the modulation of ionic channels and consequent membrane hyperpolarization^9,10^. Such hyperpolarization could be consecutive to TREK-1 activation as it is largely expressed in the myocarde^11,12^. TREK channels are mechanosensitives, and reconstituted TREK-1 and TRAAK channels in proteoliposomes exhibit a mechanosensitive gating only meadiated by the lipid bilayer, in the absence of all other cellular components^13^. Since TREK-1 and TRAAK are also activated by arachidonic acid in proteoliposomes, the gating of TREK channels by PUFAs could occurs in principle either trhough the lipid bilayer or through a direct interaction but for tether-mediated mechanism^13^. The comparison of the effects of different PUFAs on TRAAK^14^ suggests that PUFAs can insert inside the membrane, consequently inducing mechanical stress^15,16^ around the channel. While it is known that PUFAs also activate TREK-1 channel, no study has compared the effects of different PUFAs on TREK-1 to determine a potential mechanism of action.

Hence, the aim of this study was to perform a thorough comparison of the effects of 9 PUFAs (from 18 to 22 carbons with 2 to 6 double bonds) and 2 other C18 FA (mono-unsaturated and saturated) on the TREK-1 current (I_TREK-1_).

## Results

### TREK-1 channel activation by PUFAs is independ of acyl chain length

**Figure 1:**
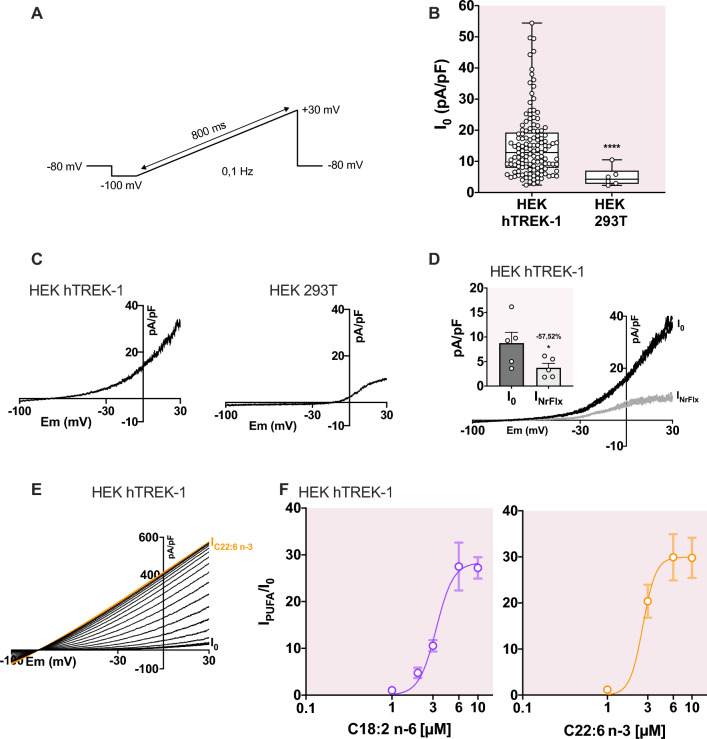
Activation of TREK-1 channel by PolyUnsaturated Fatty Acids. (**A**) Illustration of the voltage ramp protocol to record I_TREK-1_ between -100 mV and + 30 mV. (**B**) Initial current density in HEK hTREK-1 cells and in HEK 293 T cells. Groups were compared with a Mann–Whitney test. (**C**) Representative traces of initial current in HEK hTREK-1 cells (left) and in HEK 293 T cells (right) between −100 mV and + 30 mV. (**D**) Effect of 10 µM of Norfluoxetine (NrFlx) on I_0_ in HEK hTREK-1 cells. (**E**) Representative traces of current recordings during C22:6 n-3 (10 µM) application is shown in one trace *per* 10 s. (**F**) Dose–response curves of the TREK-1 activation by C18:2 n-6 and C22:6 n-3 PUFAs in the range of 1 µM to 10 µM.

In order to evaluate the TREK-1 activation by PUFAs, we used a cell line overexpressing TREK-1 channel (HEK hTREK-1 cells)^17^. Using the patch-clamp technique in whole-cell configuration, we recorded currents from a ramp protocol from -100 mV to + 30 ms, as depicted in Fig. 1A. The initial TREK-1 current density of HEK hTREK-1 cells was 15.2 $\pm$ 0.9 pA/pF at 0 mV and varied between 2.3 pA/pF and 54.4 pA/pF (n = 130) while the current density in HEK 293 T cells was 4.9 $\pm$ 0.9 at 0 mV, varying between 2.3 pA/pF and 10.5 pA/pF (n = 6) (Fig. 1B). The initial current of HEK hTREK-1 cells showed a characteristic outward rectification (Fig. 1C, left) which is not observed in HEK 293 T cells (Fig. 1C, right). As espected, the initial TREK-1 current (I_0_, black line) was inhibited by 10 µM of norfluoxetine (NrFlx). I_0_ was significantly decreased from 8.8 ± 2.2 pA/pF to 3.7 ± 0.9 pA/pF (*p*-value = 0.02; n = 5) when norfluoxetine was applied and the outward rectification was lost (Fig. 1D). Thus, I_0_ was carried out mostly by TREK-1 channel.

**Figure 2:**
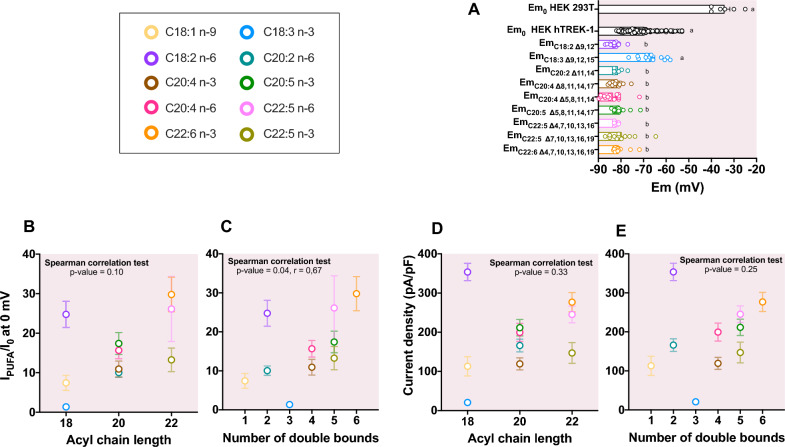
Lack of relationship between TREK-1 activation and acyl chain length. (**A**) Bar graph showing the initial membrane potential Em_0_ (mV) for HEK 293 T and HEK hTREK-1 cell lines and the membrane potential Em_PUFA_ of HEK hTREK-1 cells after the 10 µM PUFA perfusion. Groups were compared with a Kruskal–Wallis test followed with the *post-hoc* Dunn’s test. (**B)** Lack of correlation between the acyl chain length and I/I_0_ (Spearman correlation test : *p*-value 0.10, r = 0.58). (**C**) Positive correlation between the number of double bounds and the current density (pA/pF), in response to 10 µM PUFAs at 0 mV (Spearman correlation test : *p*-value 0.04, r = 0.67). (**D**) Lack of correlation between the number of double bounds and I/I_0_ (Spearman correlation test : *p*-value 0.33, r = 0.36) and (**E**) the current density (pA/pF), in response to 10 µM PUFAs at 0 mV (Spearman correlation test : *p*-value 0.25, r = 0.40).

As previously described, TREK-1 channel is activated by PUFAs, such as arachidonic acid (AA, C20:4 n-6)^7^, LA^8^ or DHA^18^. During PUFA superfusion, the current progressively increased until a steady-state (Fig. 1E). In HEK 293 T cells, there was no PUFA-activated conductance, even after 3 min of perfusion. In HEK hTREK-1 cells, we performed dose–response experiments with the shorter and the longer PUFAs, respectively C18:2 n-6 and C22:6 n-3 (Fig. 1F). The effect of PUFAs reached a maximum at 6 µM (Fig. 1F) thus we performed all the whole-cell experiments at 10 µM. The initial average membrane potential (Em_0_) of HEK hTREK-1 cells was −69.2 ± 0.7 mV (n = 130) compared to the average Em_0_ of HEK 293 T cells of −34.2 ± 2.4 mV (n = 6 ; Fig. 2A).

To investigate the importance of the acyl chain length and double bounds in TREK-1 activation, we compared the response of 9 PUFAs with various chain lengths, from 18 to 22 carbons with different numbers of double bounds, from 2 to 6 unsaturations and one mono-unsaturated fatty acids (C18:1 n-9) (Table 1). When TREK-1 is activated by PUFAs, Em hyperpolarized to −81.7 ± 0.3 mV (Fig. 2A, n = 130), close to the theorical equilibrium potential of K^+^ ions (E_K_ = −86.5 mV). TREK-1 was not activated by the C18:3 n-3 PUFA and consequently Em did not change (Fig. 2A).We plotted the relationship between TREK-1 current density at 0 mV in the presence of unsaturated fatty acids (I_PUFA_) normalized to the initial current density (I_0_) (this normalized parameter corresponds to the current fold-increase (I_PUFA_/I_0_)) and the number of carbons on the acyl chain. As shown in Fig. 2B, there is no statistical correlation between unsaturated fatty acid activation I_PUFA_/I_0_ and the acyl chain length (Spearman correlation test : *p*-value = 0.10), as for the current density of TREK-1 channel after PUFA perfusion (Spearman correlation test : *p*-value = 0.33) (Fig. 2D). We then plotted the relationship between I_PUFA_/I_0_ at 0 mV or current density in response to PUFA perfusion and the number of double bounds in the acyl chains and observed a positive correlation between the number of double bounds and the I_PUFA_/I_0_ (Fig. 2C, Spearman correlation test : *p*-value = 0.04, r = 0.67). However, this potential relationship was not confirmed by the simple linear regression test (*p*-value = 0.12). Moreover, no correlation was observed between the number of double bounds in the acyl chains and I_PUFA_ (Fig. 2E; Spearman correlation tests: *p*-value = 0.25). These statistical analysis reveal that there is at least no relationship between the effect of unsaturated fatty acids on TREK-1 and the acyl chain length and that a potential relationship existing with the number of double bonds requires further investigations. Accordingly, the most potent activators were both one of the shortest one, C18:2 n-6 (I_PUFA_/I_0_ = 24.8 ± 3.3; current density = 353.7 ± 22.4 pA/pF), and one of the longest one, C22:6 n-3 (I_PUFA_/I_0_ = 29.8 ± 4.4; current density = 276.8 ± 24.5 pA/pF). Conversely, C18:2 n-6 is one of the most potent activator but C18:3 n-3 failed to activate TREK-1 channel (I_PUFA_/I_0_ = 1.4 ± 0.1; Current density = 20.7 ± 4.2 pA/pF). Like C18:3 n-3, the saturated stearic acid (C18:0) had no effect on TREK-1 while the mono-unsaturated C18:1 n-9 produced a 7.4 ± 1.9-fold increase of I_TREK-1_ (n = 12, Table 1). However, statistical analysis failed to discriminate the PUFA’s effects (I_PUFA_/I_0_ parameters compared with a nonparametric kruskall-wallis test) probably due to the important variability of the effects.

**Table 1:** Fatty acids nomenclature, characteristics and effects on the TREK-1 current in whole-cell configuration of patch-clamp.

	IUPACName		Tail length	Unsaturation number	ω	I_PUFA_/I_0_	I_PUFA_(pA/pF)
C18:0	Stearic acid	18:0	18	0		1.0 ± 0.1	18.4 ± 0.5
C18:1_n-9_	Oleic acid (9)	18:1Δ9	18	1	ω-9	7.4 ± 1.9	113.1 ± 24.6
C18:2_n-6_	Linoleic acid (9, 12)	18:2Δ9,12	18	2	ω-6	24.8 ± 3.3	353.7 ± 22.4
C18:3_n-3_	Α-Linolenic acid (9, 12, 15)	18:3Δ9,12,15	18	3	ω-3	1.4 ± 0.1	20.72 ± 4.2
C20:2_n-6_	Eicosadienoic acid (11,14)	20:2Δ11,14	20	2	ω-6	10.1 ± 1.2	166.1 ± 16.2
C20:4_n-3_	Eicosatetraenoic acid (18,11,14,17)	20:4Δ8,11,14,17	20	4	ω-3	10.9 ± 2.0	119.2 ± 15.5
C20:4_n-6_	Eicosatetraenoic acid (5,8,11,14)	20:4Δ5,8,11,14	20	4	ω-6	15.7 ± 2.1	199.5 ± 23.1
C20:5_n-3_	Eicosatetraenoic acid (5,8,11,14,17)	20:5Δ5,8,11,14,17	20	5	ω-3	17.4 ± 2.8	211.7 ± 21.0
C22:5_n-6_	Docosatetraenoic acid (4,7,10,13,16)	20:5Δ4,7,10,13,16	22	5	ω-6	26.1 ± 8.3	245.3 ± 21.4
C22:5_n-3_	Docosatetraenoic acid (7,10,13,16,19)	20:5Δ7,10,13,16,19	22	5	ω-3	13.3 ± 2.9	147.1 ± 26.6
C22:6_n-3_	Docosatetraenoic acid (4,7,10,13,16,19)	20:6Δ4,7,10,13,16,19	22	6	ω-3	29.8 ± 4.4	276.8 ± 24.5

Values of I_PUFA_/I_0_ and I_PUFA_ are expressed as mean ± SEM.

### The variability in PUFA responses is related to the variable initial current density

Despite an important number of cells, we observed a large variability of the TREK-1 current activation by PUFAs. The severity of the inclusion criteria (see Material and Methods section) suggests that the variability observed in PUFAs responses is inherent to TREK-1 channel.

**Figure 3:**
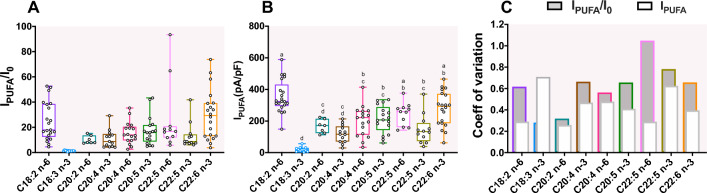
Variability of TREK-1 activation by PUFA (**A**) Boxplot of the fold increase (I_PUFA_/I_0_) of TREK-1 and (**B**) the current density at steady-state (I_PUFA_, pA/pF) at 0 mV for each PUFA at 10 µM. Boxplots represent the median (lines) with max and min values (error bars). Groups were compared with a Kruskal–Wallis test followed with the *post-hoc* Dunn’s test. Two bars having the same letter are not significantly different. (**C**) Changes in the coefficient of variation (SD/mean) between I_PUFA_/I_0_ and I_PUFA_ at 0 mV. (white : coefficient of variation for I_PUFA_/I_0_; grey: coefficient of variation for I_PUFA_). Both coefficient of variation are superimposed.

**Figure 4:**
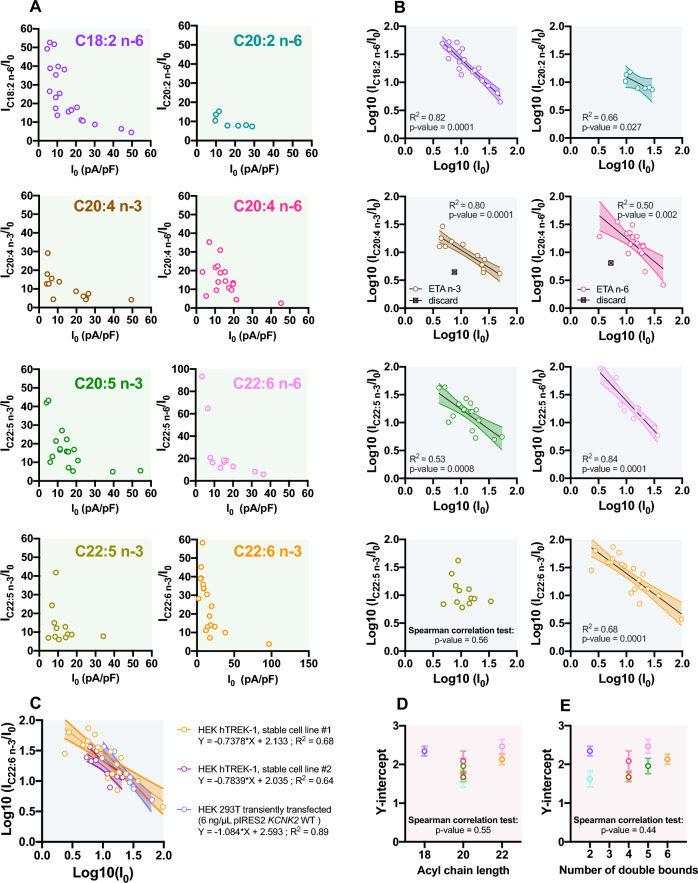
Variability in TREK-1 activation by PUFAs depends on the initial current I_0_. (**A**) Scatter plot representations of I_PUFA_/I_0_ as a function of I_0_. (**B**) Linearization of the I_PUFA_/I_0_ *vs* I_0_ relationship following log-transformation for each PUFA. The simple linear regressions (line) are represented with the 90% confidence interval (IC; dotted line) used to discard one of the C20:4 n-3 and C20:4 n-6 data points (boxes). A point is discarded only if it is out by more than twice the 90% IC. (**C**) Relationship between Log10(I_C22:6 n-3_/I_0_) and Log10(I_0_) at 0 mV in different cell lines: HEK hTREK-1 stable cell line #1 (orange), HEK hTREK-1 stable cell line #2 (red) and HEK 293 T cells transiently transfected with TREK-1 channel (pIRES2 KCNK2 WT 6 ng/µL)(purple). (**D**) Lack of correlation between the Y-intercept of the linear regressions obtained before and the acyl chain length, (**E**) and the number of double bounds, for each PUFA at 10 µM.

Figure 3A illustrates the large variability of PUFA effects based on the fold-increase analysis (I_PUFA_/I_0_). Te variability of the PUFA-induced current (I_PUFA_) appeared less wide (Fig. 3B). Indeed, the calculation of the coefficient of variation (CV = $\frac{SD}{Mean}$) indicated that the dispersion of the I_PUFA_/I_0_ calculation is higher than the dispersion of the I_PUFA_ at steady-state of the activation (Fig. 3C). Thus, CV modification is in accordance with the hypothesis that the variability of I_0_ might be responsible of the variability of the I_PUFA_/I_0_ parameter. We demonstrated that the variability of the I_PUFA_/I_0_ parameter did not resulted from the variability of current density at steady-state after the application of PUFAs (I_PUFA_) (Supplementary Fig. 1) but from the variability of the initial current I_0_ (Fig. 4A,B).

To better characterize the relationship between the effects of PUFAs and I_0_, we plotted “fold-increase of TREK-1 current” = f(“initial current”)_._ As shown in Fig. 4A, there is a non-linear relationship between I_PUFA_/I_0_ and I_0_. This negative relationship can be linearized by log-transforming the data (Log10) (Fig. 4B, Table 2). Thus, the effects of all PUFAs but C22:5 n-3 depends on I_0_, independently of the absolute amplitude of I_PUFA_. This inverse relationship indicates that the variability of I_0_ is not mainly due to a variable level of TREK-1 expression. To determine if the observed variability is linked to the cellular model that we used, or not, we also performed some experiments on two other models: another stable model of TREK-1 overexpression^19^ and transiently transfected HEK 293 T cells with TREK-1 (pIRES2 *KCNK2* WT) (Fig. 4C). The I_PUFA_/I_0_ variability observed can be explained by the variety of constitutively active TREK-1 channels in resting condition.

**Table 2:** Parameters of the linear regression Log10 (I_PUFA_/I_0_) = f(Log10(I_0_)). TREK-1 current was recorded in whole-celle configuration of patch-clamp.The p-value indicates the significativity of the relationship, the R^2^ indicates the goodness of the fit and the Y-intercept reflects the fold-activation of TREK-1 current for an unitary current as Log(1) = 0.

	n	*p*-value	R^2^	Equation	Y-intercept ± SD
C18:2 n-6	20	0.0001	0.82	Y = −0.9378*X + 2.343	2.3 ± 0.1
C20:2 n-6	7	0.027	0.66	Y = −0.5264*X + 1.621	1.6 ± 0.2
C20:4 n-3	12	0.0001	0.80	Y = −0.6305*X + 1.672	1.7 ± 0.1
C20:4 n-6	16	0.002	0.50	Y = −0.8315*X + 2.083	2.1 ± 0.3
C20:5 n-3	17	0.0008	0.53	Y = −0.7172*X + 1.958	1.9 ± 0.2
C22:5 n-6	11	0.0001	0.84	Y = −1.073*X + 2.462	2.5 ± 0.2
C22:5 n-3	12	0.371	0.08	Y = −0.3221*X + 1.378	1.4 ± 0.4
C22:6 n-3	20	0.0001	0.68	Y = −0.7378*X + 2.133	2.1 ± 0.1

By linear regression analysis, we determined the Y-intercept which reflects the fold-activation of an unitary current TREK-1 current (Log(1) = 0). The Y-intercept of DPA n-3 cannot be calculated since there was no correlation between I_PUFA_/I_0_ and I_0._ For the 7 others PUFAs, the Y-intercept values allow their separation into 3 groups with the following activation sequence : C22:6 n-3, C22:5 n-6, C18:2 n-6 > C20:5 n-3, C20:4 n-6 > C20:4 n-3, C20:2 n-6 (Table 2). Then, plotting the relationship between Y-intercept and the number of carbons revealed no correlation between the fold-activation of TREK-1 and the acyl chain length of PUFAs (Fig. 4D) or with the number of double bounds (Fig. 4E). In conclusion, TREK-1 activation by PUFAs is not dependent of the acyl chain length and the number of double bond (Fig. 2B–E and Fig. 4D,E).

### Variable activation rates suggest different PUFA binding affinities for TREK-1 channel

**Figure 5:**
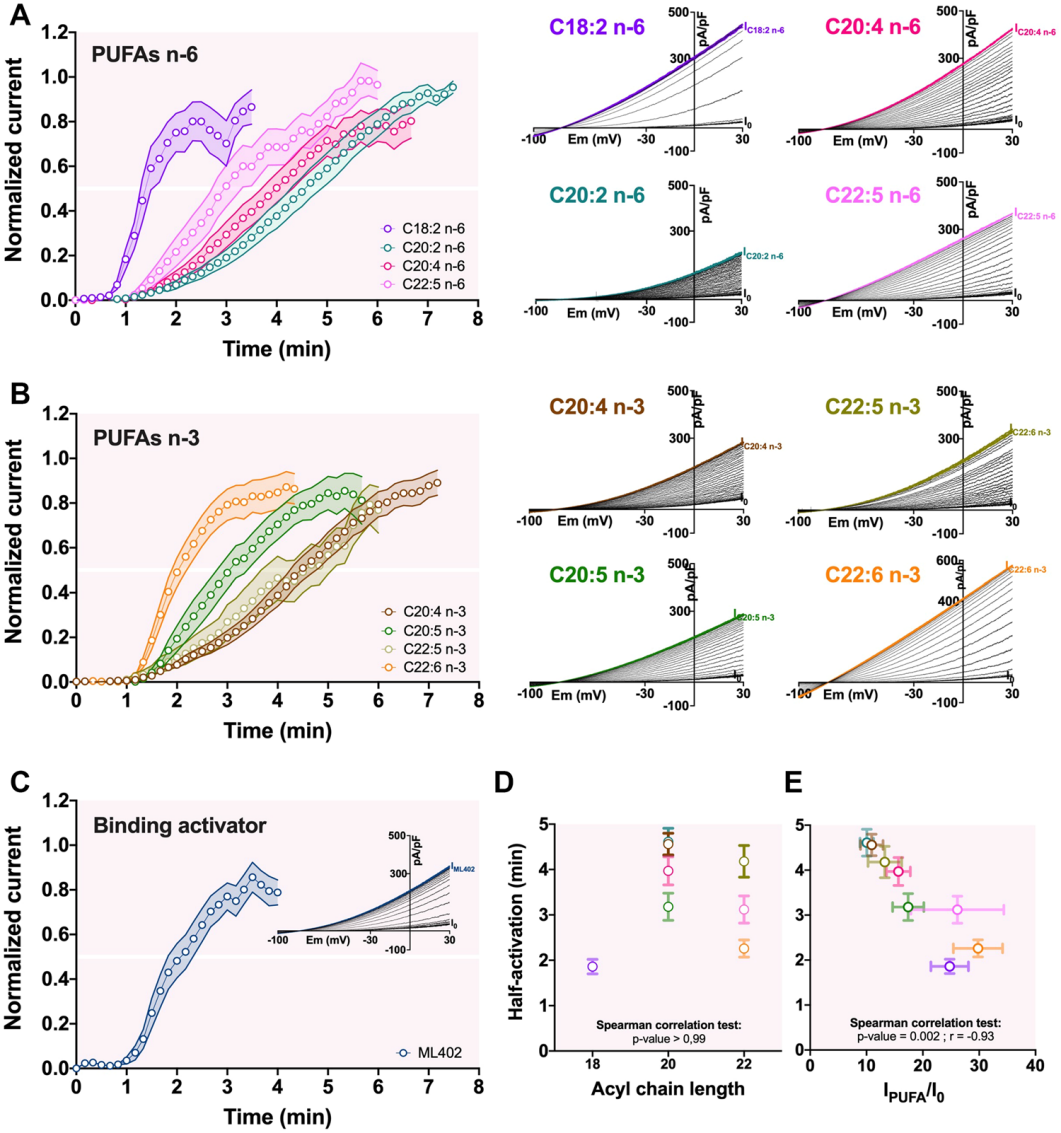
Activation kinetics of TREK-1 channel by PUFAs. (**A**–**C**) Time course showing the effects of 10 µM PUFAs and ML402 on I_TREK-1_ at 0 mV. PUFAs and ML402 were superfused until the steady-state was reached. *Insets* show the representative current densities recorded in control medium and then under PUFAs or ML402 application. (**D**) Lack of correlation between the half-activation and the acyl chain length of PUFAs (Spearman correlation test : *p*-value > 0.99). (**E**) Relationship between the fold-increase of TREK-1 (I_PUFA_/I_0_) and the half-activation (min) (r = −0.93; *p*-value = 0.002; error barres show the SEM of the I_PUFA_/I_0_ and the half-activation).

To explore a new mode of action of PUFAs on TREK-1 channel, we compared the kinetics of activation of TREK-1 perfusing PUFAs or ML402, a binding activator of TREK-1. Figure 5A–C represent the mean ± SEM of the normalized current densities ($\frac{I-{I}_{0}}{{I}_{PUFA}-{I}_{0}}$) over the time in response to PUFA and ML402. A steady-state of activation was reached when 3 successive traces were stable. The half-activation of TREK-1 by each PUFA and ML402 are presented in the Table 3. We were able to distinguish at least two types of activation rate, a fast one with an half-activation less than 3 min (C18:2 n-6, C22:6 n-3 and ML402) and a slower one above 3 min (C20:2 n-6, C20:4 n-3, C20:4 n-6, C20:5 n-3, C22:5 n-6 and C22:5 n-3). The Fig. 5D shows no statistical correlation between the acyl chain lengh, and thus their ability to incorporate into the membrane, and the half-activation (Spearman correlation test : *p*-value > 0.99). As reported in Table 3, the averaged half-activation of TREK-1 channel was lower for C18:2 n-6 and C22:6 n-3, both having comparable kinetics to those observed for ML402. Although there were no significant differences between these three compounds and C22:5 n-6 and C20:5 n-3, the kinetics of the latters appeared slower (Fig. 5A,B and D, Table 3). In contrast, C20:4 n-3, C20:4 n-6 and C22:5 n-3 had slower kinetics with an averaged half-activation close to 4 min (Table 3). Since C18:2 n-6 and C22:6 n-3 have the same fast activation and C22:5 n-3 has the slowest one, we assumed that the activation rate of the TREK-1 by PUFAs does not depend on the acyl chain length (Fig. 5D). However, among the PUFAs, there is a negative correlation between the half-activation (min) and the fold-increase of TREK-1 current (I_PUFA_/I_0_) (Spearman correlation test : *p*-value = 0.002 and r = −0.93; Fig. 5E). As the stronger activators are the faster activators of TREK-1, we propose that some PUFAs, such as C18:2 n-6 and C22:6 n-3, have a higher binding affinity for TREK-1 which would allow them to activate it faster and stronger.

**Table 3:** Parameters of the activation kinetic of TREK-1 by PUFAs and ML402 in whole-cell configuration of patch-clamp.

	n	Half-activation (min)	Statistical differences	Steady-state (min)
		Mean ± SEM		Mean ± SEM
C18:2 n-6	20	1.86 ± 0.16	a	3.65 ± 0.28
C20:2 n-6	7	4.61 ± 0.30	b,c	7.40 ± 0.25
C20:4 n-3	12	4.56 ± 0.24	b,c	7.13 ± 0.23
C20:4 n-6	16	3.97 ± 0.31	b,c	6.67 ± 0.34
C20:5 n-3	17	3.18 ± 0.30	b,c	5.65 ± 0.33
C22:5 n-6	11	3.12 ± 0.30	a,c	5.31 ± 0.41
C22:5 n-3	12	4.18 ± 0.35	b,c	5.95 ± 0.37
C22:6 n-3	20	2.26 ± 0.19	a	4.34 ± 0.27
ML402	19	2.11 ± 0.16	a	3.86 ± 0.33

Values indicate the half-activation time (min) and the steady-state (min) of the activation of TREK-1. The statistical comparison of the half-activation time for each PUFAs was performed with a Kruskal–Wallis test followed with the *post-hoc* Dunn’s test. Two rows having the same letter are not significantly different. Data are expressed as mean $\pm$ SEM.

### Activation of TREK-1 channel by PUFAs is fully reversible

**Figure 6:**
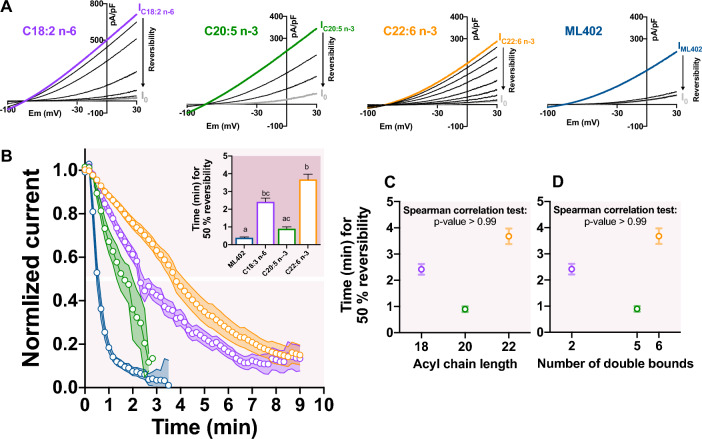
Washout kinetics of LA, EPA, DHA and ML402. (**A**) Representative traces of the reversibility of the current density with one trace per minute (grey: initial current I_0_; colors: PUFA-activated current density at steady-state; black: washout current density). (**B**) Normalized current–time curve of the reversibility for each compound (mean ± SEM) with 1 point every 10 s. The current was normalized as (I-I_0_)/(I_PUFA_-I_0_). The inset shows the time to have 50% of the effective reversibility of TREK-1 activation. (**C**) Lack of correlation between the time for 50% of effective reversibility and the acyl chain length and (**D**) the number of double bounds.

To see if the activation of TREK-1 channel by PUFA is due to an insertion and thus a modification of membrane tension, we looked at the reversibility. We focused on C18:2 n-6, C20:5 n-3 and C22:6 n-3, the most potent activators (Table 1). ML402 activation reversed immediately and 50% of effective reversibility occurred in less than 1 min (Fig. 6A,B). C20:5 n-3, had a kinetic of washing (washout time 50%: 0.9 ± 0.1 min) comparable to ML402 (Fig. 6A,B). Even though the washout of C18:2 n-6 and C22:6 n-3 was slower than ML402 (washout time 50%: 2.4 ± 0.2 min, 3.7 ± 0.3 min and 0.4 ± 0.04 min, respectively) , PUFAs effects were also fully reversed under washing. No correlation between the acyl chain length (Fig. 6C, Spearman correlation test: *p*-value > 0.99) or the number of double bounds (Fig. 6D, Spearman correlation test: *p*-value > 0.99) and the time to have 50% of effective reversibility of the TREK-1 activation was found. C18:2 n-6 and C22:6 n-3, that activated TREK-1 at least twice more than ML402 (I/I_0_: 24.8 ± 3.3, 29.8 ± 4.4 and 9.6 ± 0.9, respectively), had a total reversibility in few minutes. At this point, we cannot exclude a membrane insertion of PUFAs, but we assume that the main effects of PUFAs on TREK-1 activation could be a direct and reversible interaction of PUFAs with the channel as it is known for KCNQ1^20^ and for the Shaker H4 Kv channel^21^.

### Alteration of membrane curvature or fluidity did not explain PUFAs activation of TREK-1 channel

**Figure 7:**
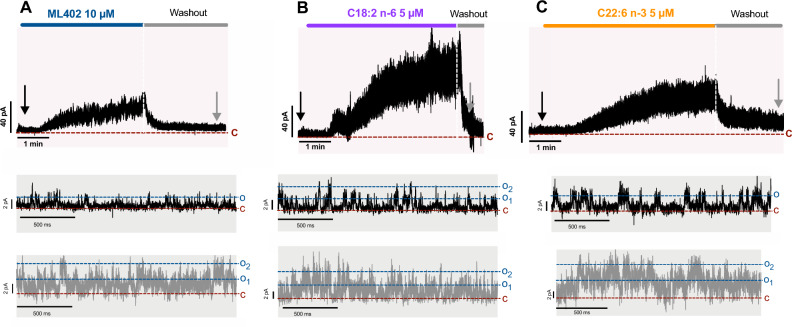
PUFAs activate TREK-1 in inside-out configuration of patch-clamp. (**A**–**C**) Representative traces showing the activation and reversibility in inside-out configuration of patch-clamp technique for (**A**) ML402 10 µM (**B**) C18:2 n-6 5 µM and (**C**) C22:6 n-3 5 µM superfused at the inner face of the membrane. Membrane potential was held at + 30 mV. The expanded current traces where extracted at the time indicated by the arrows.

In order to evaluate the membrane curvature and tension effects on the PUFAs-induced TREK-1 activation, we performed experiments in the inside-out configuration of the patch-clamp technique (+ 30 mV, symmetrical condition: 145 mM KCl). In this configuration, we were able to superfuse molecules at the inner face of the membrane and PUFAs must induce an aopposite curvature of the membrane than in the whole-cell configuration. As shown in Fig. 7A, ML402 superfusion at the inner face of the membrane reversibly increased TREK-1 current (Table 4). Kinetics of activation and reversibility were comparable to those obtained in the whole-cell configuration (Figs. 5 and 6) suggesting that the ML402-binding site is accessible from the outer and the inner membrane leaflet. Interestingly, a comparable reversible activation of TREK-1 channel was obtained for C18:2 n-6 and C22:6 n-3, 5 µM, suggesting that the membrane curvature is not involved in the activation of TREK-1 channel by PUFAs (Supplementary Fig. 2B,C, Table 4). In the other hand, the inside-out experiments showed that PUFAs activation occured independently of scaffold proteins like PLD2 known to modulate TREK-1 activity in response to disruption of lipid rafts^22,23^.

**Table 4:** Descriptive statistics for TREK-1 activation and reversibility kinetics in inside-out configuration of patch-clamp.

			Steady-state (min)	50% of reversibility (s)
	n	Concentration (µM)	Mean ± SEM	Mean ± SEM
C18:2 n-6	4	5	4.6 ± 0.4	18.3 ± 6.3
C22:6 n-3	6	5	2.9 ± 0.3	18.3 ± 4.2
ML402	6	10	2.6 ± 0.6	15.5 ± 7.6

Values indicate the steady-state time (min) and the time to have 50% of effective reversibility of TREK-1 activation (s).

Then, we assessed the membrane fluidity changes during PUFAs application with a pyrenedecanoic acid probe (PDA), analog to lipids. By measuring the ratio of PDA monomer to excimer fluorescence (405 nm/470 nm ratio), a quantitative assesment of the membrane fluidity can be obtained at different time points by following the ratio modification over time (F/F_0_-1). We focus our experiments on C18:2 n-6 and C22:6 n-3 the stronger activators of TREK-1 at 10 µM and C18:3 n-3 that failed to activate TREK-1. These 3 PUFAs at 10 µM did not modify the membrane fluidity even after 50 min of application, while at 100 µM they induced a decrease of F/F_0_-1 from t_0_ compared to the control condition (basic extracellular medium). These results indicate that membrane fluidity is not modified by PUFAs at 10 µM (Sup Fig. 2A–C), at least within 50 min of application. In addition, given that TREK-1 activation starts at 1 min of perfusion of C18:2 n-6 and C22:6 n-3 10 µM (Fig. 5) it is unlikely that PUFA effects on TREK-1 activation are due to an increase in membrane fluidity. Altogether, these data suggest that at least both C18:2 n-6 and C22:6 n-3 PUFAs activate TREK-1 channel by direct interaction with TREK-1 protein and not by a modification of the membrane fluidity. We cannot exclude that the probe used to measure membrane fluidity modification in response to PUFA is not sensitive enough in small range of concentration (*ie* 10 µM) but the sensitivity at 100 µM is a good indicator of the changes in membrane fluidity.

### DHA interacts directly with TREK-1 channel protein in TREK-1 enriched microsomes

In order to assess a potential direct PUFA-TREK-1 interaction, we purified microsomes from hTREK-1/ HEK and native HEK 293 T cells and labeled lysine residues of the total proteins to perform affinity measurements using Spectral Shift (SpS). Data of the affinity curve are expressed as mean $\pm$ SEM of 5 to 9 experiments from different batches of microsomes of “hTREK-1/ HEK” and “native HEK 293 T” cells. TREK-1 protein quantity is constant within the 16 samples from microsomes from hTREK-1/ HEK. At first glance, we observed similar affinity from C22:6 n-3 in these two type of microsomes : K_d,TREK-1_ ~ 50 µM and K_d,HEK_ ~ 100 µM highlighting a similar mode of association of C22:6 n-3 within the microsomes. However, the SpS signal displayed subtle differences according to whether or not microsomes were enriched in TREK-1 protein. Knowing SpS signal rises from fluorescence recorded by two individual channel, and having a strong reproducibility from each condition, we can derive the following assumption:

1 $${\text{I}}_{\uplambda }\left(\text{TREK}1,\text{HEK}\right)= {\text{I}}_{\uplambda }\left(\text{TREK}1\right)+ {\text{I}}_{\uplambda }\left(\text{HEK}\right)$$

where for each individual wavelength (λ_670 nm_ and λ_650 nm_), the fluorescence recorded for the TREK-1-enriched microscomes (I_λ_(TREK1, HEK)) correspond to both the fluorescence from the labelled empty microsomes (I_λ_(HEK)) and from the labelled TREK-1 (I_λ_(TREK1)).

Being able to isolate the specific fluorescence associated to TREK-1 $({\text{I}}_{\uplambda }\left(\text{TREK}1\right))$ within the TREK-1-enriched microsomes (${\text{I}}_{\uplambda }\left(\text{TREK}1,\text{HEK}\right))$, we can use individual channel for further calculation using Eq. 2, isolating the SpS signal associated only to TREK-1 by normalizing out the background signal coming for the free microsomes.

2 $$\text{R}(\text{TREK}1)=\frac{{{\text{I}}_{670}(\text{TREK}1,\text{HEK})-{\text{I}}_{670}\left(\text{HEK}\right)}}{{\text{I}}_{650}\left(\text{TREK}1,\text{HEK}\right)-{\text{I}}_{650}(\text{HEK})}$$

**Figure 8:**
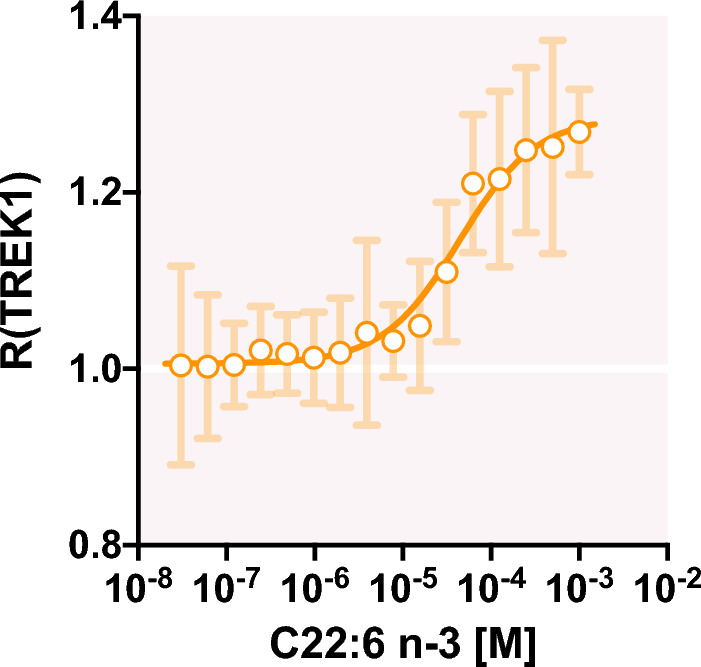
Effect C22:6 n-3 on SpS signal in TREK-1-enriched microsomes and non-enriched microsomes. (${\text{I}}_{\uplambda }\left(\text{TREK}1\right)$ represents the binding affinity of C22:6 n-3 (from 1 mM to 30.5 nM) for TREK-1-enriched microsomes from 1 mM to 30.5 nM (n = 5–9 experiments).

When performing so, the specific TREK-1 dose–response gives a K_d_ = 44 µM for C22:6 n-3 (Fig. 8). Despite displaying similar affinities, the TREK-1-enriched microsomes shows a statistically better affinity than the microsomes themselves. However, it is worth noticing only a twofold increase of affinity, which may highlight a similar binding mode for both interactions. Altogether, this suggests an interaction mediated by the lipid bilayer, such as a membrane insertion followed by interaction with TREK-1 channel.

## Discussion

### TREK-1 channel activation by PUFAs involves a direct interaction

In this study, we report that TREK-1 channel is reversibly activated by polyunsaturated fatty acids (PUFAs), as already shown in different studies, each being focused mainly on one PUFA : AA^7^ (C20:4 n-6); LA^8^ (C18:2 n-6) and DHA(C22:6 n-3)^18^. Our study is the first to compare the effects of different PUFAs having between 18 to 22 carbon atoms and 2 to 6 double bonds on TREK-1 channel. We demonstrate that C22:6 n-3 and C18:2 n-6 are the most potent activators of TREK-1 with a kinetic close to the direct activator ML402 and a fully reversibility.

Since TREK-1 channel is mechanosensitive and activated by arachidonic acid in cells^7^ and liposomes^13^ it is hypothetized that its gate is either mediated by changes in the membrane curvature induced by PUFAs insertion leading to an increase of the mechanical stress transmitted to the channels^7^ as for CPZ^7^ or by a direct binding of PUFAs. As PUFAs are anionic amphipath compounds, with a hydrophilic carboxyl group and a lipophilic tail, they preferentially insert into the outer leaflet of the membrane which is positively charged^16,24^. Thus, the longer the lipophilic carbon chain is, the more the PUFAs will be inserted into the membrane, modifying the local membrane biophysic properties (curvature and fluidity)^15^. Also, for a given carbon chain length enrichment, the membrane fluidity increases with the number of double bonds. The study of the TRAAK channel activation by PUFAs (C18:2 n-6, C20:4 n-6, C20:5 n-3, C22:6 n-3) in the excised patch configuration shows that TRAAK activation is positively correlated with the carbon chain length of PUFAs and the number of double bonds^25,26^. However, in the present study, we found no statistical correlation between the acyl chain length and the potentiation of I_TREK-1_. In the opposite, C18:2 n-6 and C22:6 n-3, respectively the shortest and the longest PUFA tested, are the most potent activators of TREK-1 channel. It is worth to note that C18:3 n-3, which differs only by one double bond from C18:2 n-6, failed to activate TREK-1 channel. The presence of this extra double bond, modifying the steric hindrance of the fatty acid, prevents TREK-1 channel activation, probably due to congestion which avoids the direct interaction with the channel. Also, C22:6 n-3 having the same number of carbons than C22:5 n-3 is more than twice as effective in activating TREK-1. Therefore, the acyl chain length does not appear to be a decisive feature in the mechanism of action of PUFAs. The other PUFAs tested produce intermediate activation of TREK-1 (between the effect of C18:2 n-6 and C22:6 n-3), in the same range as the direct activator ML402, independently of the double bonds number. This finding and the weak activation of TRAAK by C18:2n-6^25^ shows that TREK-1 behaves differently of TRAAK. In that respect, the bilayer-couple hypothesis suggesting an activation of TREK-1 by PUFAs only mediated by the lipid bilayer appears to be inappropriate. Similar results were obtained in the literature on TREK-2 channel study, C20:4 n-6 being less efficient than C22:6 n-3 and C18:2 n-6^27^.

To better characterize the mechanism of action of C22:6 n-3 and C18:2 n-6 on TREK-1, we compared their activation and reversibility with ML402. ML402 is a direct activator of TREK-1, binding within a cryptic pocket behind the selectivity filter that directly stabilize the C-type gate^28^. The activation kinetics of TREK-1 by C22:6 n-3, C18:2 n-6 and ML402 are comparable and faster than the other PUFAs. This suggests a possible interaction of C22:6 n-3 and C18:2 n-6 with the channel like the activator ML402^28^ and the inhibitor norfluoxetine^29^. Other studies showed that anionic lipids (PIP_2_, phosphatidic acid) can directly bind to and gate ion channels such as TREK-1 channel^30–32^. By competition assay, a previous study demonstrated that n-3 ETA, n-6 ETA (AA) and DHA free fatty acids (C20:4 n-3, C20:4 n-6 and C22:6 n-3) competed with PIP_2_ binding on TREK-1 channel transmembrane domain and concluded that this binding was highly specific^32^. This binding hypothesis is reinforced by the inside-out experiments where C22:6 n-3, C18:2 n-6 and ML402 were applied on the inner leaflet of the membrane. Although the PUFA insertion must induce opposite local curvartures while they insert from the inner (inside-out configuration) or the outer leaflet (whole-cell configuration), they still activate TREK-1 channel. We finally propose that at least C22:6 n-3 and C18:2 n-6, like ML402, interact with the channel on lipid binding site(s) accessible from both the inner and the outer leaflet of the cell, possibly through the lipid bilayer. Studies have already hypothesized that arachidonic acid (C20:4 n-6) could act directly by interacting with the channel^33,34^ and others have already shown that free PUFAs can directly interact with ion channels. Indeed, C22:6 n-3 and C18:3 n-6 interact with K_v_7.1 (*KCNKQ1*)^20,35^. PUFAs also interact with Shaker H4 Kv channel closed to the voltage-sensor domaine through the negatively charged carboxyl group^21,36,37^. As TREK-1 lacks a canonical voltage-sensor domain, we can hypothesize that there is another lipophilic binding site in TREK-1 interacting with the carboxyl head of PUFAs.It has to be noted that bovine serum albumin (BSA) is not required to get a full reversibility during the washout as it is supposed to be when the effects are due to membrane insertion of the PUFAs^9,15^. The structure of TREK-1 channel is now known since 2017^28^. Structural studies revealed multiple binding sites for modulators of TREK-1 activity at every layer of the protein from the extracellular side (loop connecting the P1 helix to the CAP domain) to the intracellular side (C-terminal tail), even in the portion of the channel that interacts with the membrane bilayer^38,39^. As for all the K2P channels, its principale gate is the “C-type” gate of selectivity filter. ML402 activator acts at the L-shaped pocket found in the P1-M4 interface to stabilize it and activates the selectivity filter C-type gate^28^. However, the PUFA binding site on TREK-1 does not correspond to the binding site of ML402 since its mutation does not prevent activation by C20:4 n-6^28^. The fenestration site open to the center of the membrane bilayer below the P2 helix could represent a coherent binding site for PUFAs^38^. Its position to the center of the membrane makes this fenestration binding site accessible from both the inner and the outer leaflets of the cell consistent with the reversible activation of TREK-1 by PUFAs in inside-out and whole-cell configurations. In these both configurations of the patch-clamp technique, the reversibility of PUFAs effects were immediate and the initial current recovered in few minutes. Despite a total reversibility of the TREK-1 activation under washout and owing to their lipophilic properties, we do not exclude that a membrane insertion of PUFAs is required to reach the binding site. More recently, using cryo-EM method, Schmidpeter et al*.* demonstrated that anionics lipids bind to the extracellular side of TREK1, inserting the hydrocarbon tail into a pocket behind the selectivity filter, causing a structural rearrangement knowing to activate TREK-1^40^.

Affinity measurements between PUFAs and TREK-1-enriched microsomes effectively indicates a potential binding with the lipid bilayer as observed for the empty microsomes. However, once the signal due to PUFA interaction with the bilayer is subtracted from the total signal, a direct binding of PUFAs to TREK-1 is measurable. This specific interaction displays a stronger affinity (K_D,TREK-1_ $\sim$ 44 µM) than the simple signal involving PUFA binding with the bilayer of the microsomes. Therefore, we propose two mechanisms acting together to increase I_TREK-1_: (1) PUFA insertion into the membrane, with no modification of its biophysic properties over few minutes at 10 µM concentration or less, is probably recquired to reach (2) a lipophilic binding site on TREK-1 channel accessible through the lipid bilayer as for the Shaker channel^37^.

### Initial TREK-1 variability influences PUFAs response

An unexpected result of this study is the observation that the PUFA effects depend on the TREK-1 initial current. A variability in TREK-1 expression between HEK hTREK-1 cells cannot explain the variability of the PUFA effects since the amplitude of the TREK-1 activation (I/I_0_) varies in an inverse proportion to the initial TREK-1 activity (I_0_) rather than proportionally. This variability in initial I_TREK-1_ might have many origins such as different levels of post-translationnal modifications, different channels recruitment into the membrane or even the presence of 2 different conductances of TREK-1 channel at the single channel level^19,41^. A comparable variability in the current-fold increase induced by PUFAs was already observed for TREK-1 activation by AA^34^, TRAAK^25,26^ and TREK-2^27,42^ but never explained.

All experiments were performed on COS-7 cells or HEK-293 cells where mammalian post-translational modifications exist. In the study of Ma and Lewis in 2020, whole-cell recording of TREK-1 and TREK-2 by arachidonic acid were performed in oocytes (*Xenopus Laevis*) and no such variability in the current was observed. It is known that in this model, post-translational modifications are differents^43^. Therefore, the possibility that constitutive TREK-1 current varies accordingly with the phosphorylation level (or other modifications) and influence the effects of PUFAs should be taken in consideration. TREK-1 channel is modulated by intracellular signalling pathways, related to PKA, PKC and PKG signalizations^44–46^, but to the best of our knowledge, no study between PUFA activation of TREK-1 and phosphorylation levels has been performed so far. Consequently, to study the effect of an activator of I_TREK-1_, we need a sufficient number of cells to apply the Y-intercept of the regression line, probably the best indicator of the degree of activation of TREK-1. The existence of such variability should be taken into consideration in pathophysiological studies associated with variability of expression or response, potentially demultiplying the heterogeneity of TREK-1 response to activators.

Surprisingly, one of the most efficient activator of TREK-1 channel is C18:2 n-6. As TREK-1 is expressed in cardiomyocytes^1,4,47^ C18:2 n-6 should modulate action potential shape and resting membrane potential.This suggests that C18:2 n-6 could display potent cardio as well as neuroprotective effects. If anti-arrhythmic properties of omega-3 PUFAs have been thoroughly studied^9,10,48^, the potential anti-arrhythmic effect of omega-6 PUFAs such as C18:2 n-6 has never been considered. C18:2 n-6 can be found in vegetarian and Mediterranean diets, both diets having cardioprotective vertues^49,50^. In both cases the beneficial effects are attributed to the presence of anti-oxidant molecules and C18:3 n-3 but a possible beneficial effect of C18:2 n-6 has never been explored.

## Conclusion

To conclude, there is no relationship between the PUFA carbon or double bounds number and the activation of TREK-1 channel. Its most potent activators are C18:2 n-6 (LA) and C22:6 n-3 (DHA) and kinetics analysis suggest a direct interaction of PUFAs on TREK-1 through a lipophilic binding site. This direct activation of PUFAs in TREK-1 could require the membrane insertion of PUFAs to facilitate the access to the binding site in the channel.

## Materiel and methods

### Cell culture

We used a HEK-293 T cell line for spectral shift mesurement and two HEK/hTREK-1 cell lines that stably overexpress the human TREK-1 channel subunit^17,19^ for electrophysiological experiments and spectral shift mesurement. Cells were grown in an atmosphere of 95% air/5% CO_2_ in Dulbecco’s modified Eagle’s medium and Glutamax (Invitrogen, Cergy- Pontoise, France) supplemented with 10% (v/v) heat inactivated fetal bovine serum and 0.5 mg/mL G418 to maintain a selection pressure in the HEK/hTREK-1 cell line.

The transient expression of TREK-1 was performed in the HEK 293 T cell line. The pIRES2 plasmid in which the coding sequence of TREK-1 (pIRES2 *KCNK2* WT) was inserted, was transfected in HEK 293 T cells using the jetPEI® kit (Ozyme) and following the manufacturer protocol. Briefly, 48 h before electrophysiological experiments, cells were transfected with a mix of the water-soluble polymer jetPEI® with DNA at 6 ng/mL and then seeded in 35 × 10 mm dishes (Falcon) in presence of 0.5 mg/mL of G418.

### Electrophysiology

Currents were recorded from hTREK-1/HEK-cells using the patch-clamp technique in whole-cell recording (WCR) configuration and in inside-out (IO) configuration. Patch pipette having 2.5–4 MΩ resistances (WCR) and 5–7 MΩ resistances (IO) were obtained from borosilicate glass capillaries by using a two-stage vertical puller (PC- 10, Narishige, London, UK). Current acquisition was performed with an Axopatch 200B amplifier (Axon Instrument, Sunnyvale, CA, USA) and low-pass filtered at 5 kHz (WCR) and 2 kHz (IO). Data were digitalized with a digidata 1550B (Axon Instrument, Sunnyvale, CA, USA) at 20 kHz. The pClamp10.7 (Axon Instrument) software was used to impose stimulations protocols and record TREK-1 current. In WCR, cells were kept for experiments if the series resistance were lower than 8 MΩ, the membrane capacitance between 20 and 35 pF.

The experiments were performed at room temperature (~ 22 °C). Cells were continuously superfused with a extracellular medium and modulatory compounds at a rate of 1–1.5 mL/min. The dishes volume was kept constant at 2.5–3 mL using a vacuum system, connected to a peristaltic pump (ISMATEC). Using different extracellular potassium concentrations (5 mM KCl or 15 mM KCl), changes in membrane potential were measured in current-clamp allowing to determine the time to change completely the medium around the patched-cells^51^. A complete change of the extracellular medium around the patched-cell occurred in less than 15 s.

#### Whole-cell recording

The extracellular medium consisted of (in mM): 150 NaCl, 5 KCl, 3 MgCl_2_, 1 CaCl_2_ and 10 HEPES pH adjusted to 7.4 with NaOH. The pipette solution consisted of (in mM): 155 KCl, 3 MgCl_2_, 5 EGTA and 10 HEPES, pH adjusted to 7.2 with KOH. Cells were clamped at a holding potential of −80 mV and superfused with the extracellular medium for over 1 min before the initial current (I_0_) recording. Cells were hyperpolarized to −100 mV for 50 ms and then the macroscopic outward TREK-1 current was elicited with an 800 ms voltage ramp protocol from −100 mV to + 30 mV every ten seconds (see the ramp protocole in Fig. 1A). Thus, the evolution of the current amplitude changes during compound superfusion was followed in real time (Fig. 1E). A minimum of 3 min superfusion was applied even when the tested compound showed no effect and the superfusion condition was switched once a steady-state was reached. In the case where the tested compound had no effect, a positive control of the TREK-1 activation was performed with 10 µM docosahexaenoic acid (DHA, C22:6 n-3).

#### Data analysis (WCR)

Once a steady-state was reached, the current amplitude at 0 mV during the voltage ramp was measured from the average of the last 3 sweeps over a delta of 1 mV (−0.5 mV to 0.5 mV) to get out of the noise. Current amplitudes are expressed in current densities (pA/pF) to remove the variability due to cell size. Activation an washout kinetics were followed at 0 mV.

#### Inside-out configuration

Bath medium contained (in mM): 140 NaCl, 4.8 KCl, 1.2 MgCl_2_, 10 glucose, 10 HEPES, pH adjusted to 7.4 with NaOH. After excising the membrane patch, the bath medium was replaced by the a medium identical to the pipette medium consisted of (mM): 145 KCl, 1.2 MgCl_2_, 10 glucose, 10 HEPES, pH adjusted to 7.2 with KOH. Cells were clamped at a holding potential of 0 mV, the theorical equilibrium potential for K^+^ ions in this condition. Then, the amplitude current was followed at + 30 mV in real time during the perfusion of compounds and the washout.

### Membrane fluidity experiments

Membrane fluidity was assessed using the pyrenedecanoic acid probe (PDA), a probe analog to lipids that incorporates into the cell membrane. The probes in the membrane form monomers and excimers, with a rate of excimer proportional to the membrane fluidity. Under PUFAs (C18:2 n-6, C18:3 n-3, C22:6 n-3) application at 10 µM and 100 µM, we measured the emission spectrum of the PDA: 470 nm for the excimers and 400 nm for the the monomers. The monitoring of the fluorescent ratio 470/400 nm shift over time with PUFAs allowed a quantitative monitoring of the membrane fluidity changes due to PUFAs insertion into the membrane. Briefly, HEK/hTREK-1 cells were grown in culture in glass bottom culture dishes (MatTek, Ashland, U.S.A). Before fluorescent experiment, adherent HEK/hTREK-1 cells were incubated 1 h at room temperature in the dark with 5 µM of the fluorescent lipid reagent probe in buffer provided in the membrane fluidity kit (Abcam). After incubation, the unicorporated probes were removed by washing cells twice with the same extracellular medium that used for WCR experiments. Epifluorescence microscopic experiments were performed on a 40X lens using excitation light filter with a 370-mm dichroic filter (Zeiss). Emitted light were taken at 480 ± 15 nm and 405 ± 10 nm and the 480/405 nm ratio was calculated at intial time before PUFA application (T_0_) and T_2_ (2 min), T_4_, T_6_, T_8_, T_10_, T_20_, T_30_, T_40_, T_50_ and T_60_. The 470/400 nm ratio was calculated as the average ratio overs 5 s. Then, the 470/400 nm ratio was normalized as (F/F_0_)-1 and represented over time (Fig. 9) with F the ratio of fluorescence corresponding to a time T_t_ and F_0_ the ratio corresponding to the T_0_.

### Microsomes preparation

Microsomes were obtained from the HEK/hTREK-1 cell line and from the HEK 293 T cell line as control. Briefly, cells were cultured in T75 flasks until confluence.Then, cells were washed with PBS twice and centrifugated 5 min at 15,000 RPM. The pellet was lysed in a 20 mM PIPES buffer (300 mM Sucrose, 20 mM PIPES, pH 7 with NaOH). Membranes were then mechanically braked up using insulin syringe. Samples were centrifuged 20 min at 10,000 g at 4 °C. Lysis protocol was repeated twice. The pellet was discarded and the supernatant containing plasma membrane and thus transmembrane proteins was ultracentifugated 1 h at 32,000 RPM at 4 °C (Optima^TM^L-90 K Ultracentrifuge, Beckamn Coulter; Rotor SW60Ti). The pellet containing microsomes was resuspended in 5 mM PIPES (300 mM Sucrose, 5 mM PIPES, pH 7.4 with NaOH) at a protein concentration adjusted to 25 mg/mL and conserved at −20 °C 2 days before the spectral shift measurment.

### Spectral shift measurement

Microsomes of HEK 293 T cell lin and HEK/hTREK-1 cell line were used to evaluate the TREK-1-PUFAs interaction with HEK 293 T microsomes as control. Briefly, the proteins contained in the microsomes were labeled on the lysine residues with a fluorescent dye (MO_L011 RED-NHS 2^nd^ generation, NanoTemper Tehnologies GmbH) following the manufacturer protocol. The 20 µL at 600 µM of dye were used to labelled 200 µL of microsomes, thus leading to a final incubation volume of 200 + 20 µL. After 20 min of incubation, the 220 µL of labeled microsomes were applied to a gravity size exclusion B-column supplied in the kit, following the manufacturer protocol until etution. Labeled microsomes were eluted with 5 × 200 µL of the 5 mM PIPES buffer. 5 fractions of 200 µL were collected in a clean Eppendorf. The first elution fraction contains the labeled microsomes while the last fractions contain more free dye. The fluorescence intensity of fraction 1, 2 and 3 were verified and fractions with a fluorescence count between 400 and 2,000, using 100% excitation power, were used for the experiment after a 10 min centrifugation at 16,000 g to avoid homogeneity problem. A serial dilution of C22:6 n-3 over 16 points following 1:1 pattern was set up and subsequently mixed with labeled microsomes and loaded into standard capillaries (NanoTemper Technologies GmbH). The total proteins concentration in microsomes was kept around 2.5 µg/mL whereas C22:6 n-3 was titrated from 1 mM to 30.5 nM. The read out was performed on a Monolith X instrument from NanoTemper Technologies GmbH. The samples were subjected to SpS and MST measurments and only SpS was analyzed due to the normalization requirments. The changes in the maxima emission wavelength following a ratiometric measurement upon protein-C22:6 n-3 complex formation was used to generate a binding curve as a function of C22:6 n-3 concentrations^52^. The data were pulled from 9 and 5 individual repeat for the TREK-enriched microsomes and from HEK 293 T microsomes respectively. Data processing and analysis were carried out using the MO.Control 3 software from NanoTemper Technologies followed by normalization as described in the results section. All the data points presenting signs of irregularity were automatically discarded upon merging the data, as documented in the software.

### Chemicals

In this study we tested one direct activator of TREK-1, ML402, one saturated fatty acid, stearic acid, one monounsaturated fatty acid, oleic acid, 9 different PUFAs having between 18 and 22 carbons and 2 to 6 double bonds (Table 1). All reagents are summarized in the reagents and tools table. 10 mM stock solutions were prepared by dissolving PUFAs in absolute ethanol, ML402 in DMSO. PUFAs and ML402 were stored at −80°. 10 µM solutions were obtained by diluting an aliquot of stock solution in the extracellular medium just before use for WCR experiments. Cells for current recordings were first superfused with extracellular medium containing ethanol at 1/1000 as a control.

**Table:** 

Reagent/resource	Source	Identifier or catalog number
Experimental model		
HEK hTREK-1 cell line	^17^	
HEK 293 T/hTREK-1 cell line	^19^	
HEK-293 T cell line		
Cell culture		
DMEM + Glutamax	ThermoFisher	31,966–021
Fetal bovine serum	Sigma-Aldrich	F7524-500ML
jetPEI®	Ozyme	POL101000053
Trypsin–EDTA	ThermoFisher	25,300–054
G418 (geneticin)	Sigma-Aldrich	4,727,878,001
Chemicals		
Linoleic acid	Sigma-Aldrich	L1376-500MG
cis-11,14-Eicosadienoic acid	Sigma-Aldrich	E3127-25MG
Oleic acid	Sigma-Aldrich	O1008
Stearic acid	Sigma-Aldrich	S4751-1G
Cis-5,8,11,14,17-Eicosapentaenoic acid	Sigma-Aldrich	E2011-10MG
Cis-4,7,10,13,16,19-Docosahexaenoic acid	Sigma-Aldrich	D2534-100MG
Alpha-linolenic acid	Sigma-Aldrich	L-039-1ML
Docosapentaenoic acid (cis-7,10,13,16,19)	Sigma-Aldrich	D-120-1ML
Cis-4,7,10,13,16-Docosapentaenoic acid	Sigma-Aldrich	18,566-10MG
Arachidonic acid	Sigma-Aldrich	A36-11-100MG
Omega-3 Arachidonic acid	Santa Cruz	sc229695
ML402	MedChemExpress	HY-104027
NanoTemper experiment		
Protein labeling kit	NanoTemper	MO-L011
Capillaries	NanoTemper	MO-K022
Monolith™ X instrument	NanoTemper	
Membrane fluidity experiment		
Membrane fluidity kit	Abcam	ab189819
Glass Bottom culture dishes	MatTek	P35G-0–14-C

### Statistical analysis

All set of experiments were performed on at least three different batches (congelation and/or passage) of cells. All descriptive statistics are displayed from Table 1, Table 2, Table 3 and 4. Statistical analysis were performed using Prism software (GraphPad Prism 9, Inc., USA). Spearman correlation test were performed and the p-value indicates the significance of the correlation and the r parameter indicates the direction of the correlation (negative or positive). For the linear regression, the p-value indicates the significance of the relationship, the R^2^ indicates the goodness of the fit an the error bars represent the 90% confidence interval. Kruskal–Wallis test followed by a post hoc Dunn’s test was used for multiple comparison of unmatched data. A p-value of 0.05 or less was considered as statistically significant. The differences between more than 2 groups are displayed by the letter code, where groups that do not share the same letter are significantly different.

### Supplementary Information

Supplementary Information.

## Data Availability

The authors declare that the data supporting the findings of this study are available within the paper. Should any raw data files be needed in another format they are available from the corresponding author: i.e. Pr Marie Demion at the following email adresse Marie.demion@inserm.fr upon reasonable request.
