# Supplementary figures and images for: Activation of hTREK-1 by polyunsaturated fatty acids involves direct interaction
Source: Sci Rep. 2024 Jul 2;14:15244. doi: 10.1038/s41598-024-66192-w (PMC11220079; doi:10.1038/s41598-024-66192-w)

Supplementary Figure 1

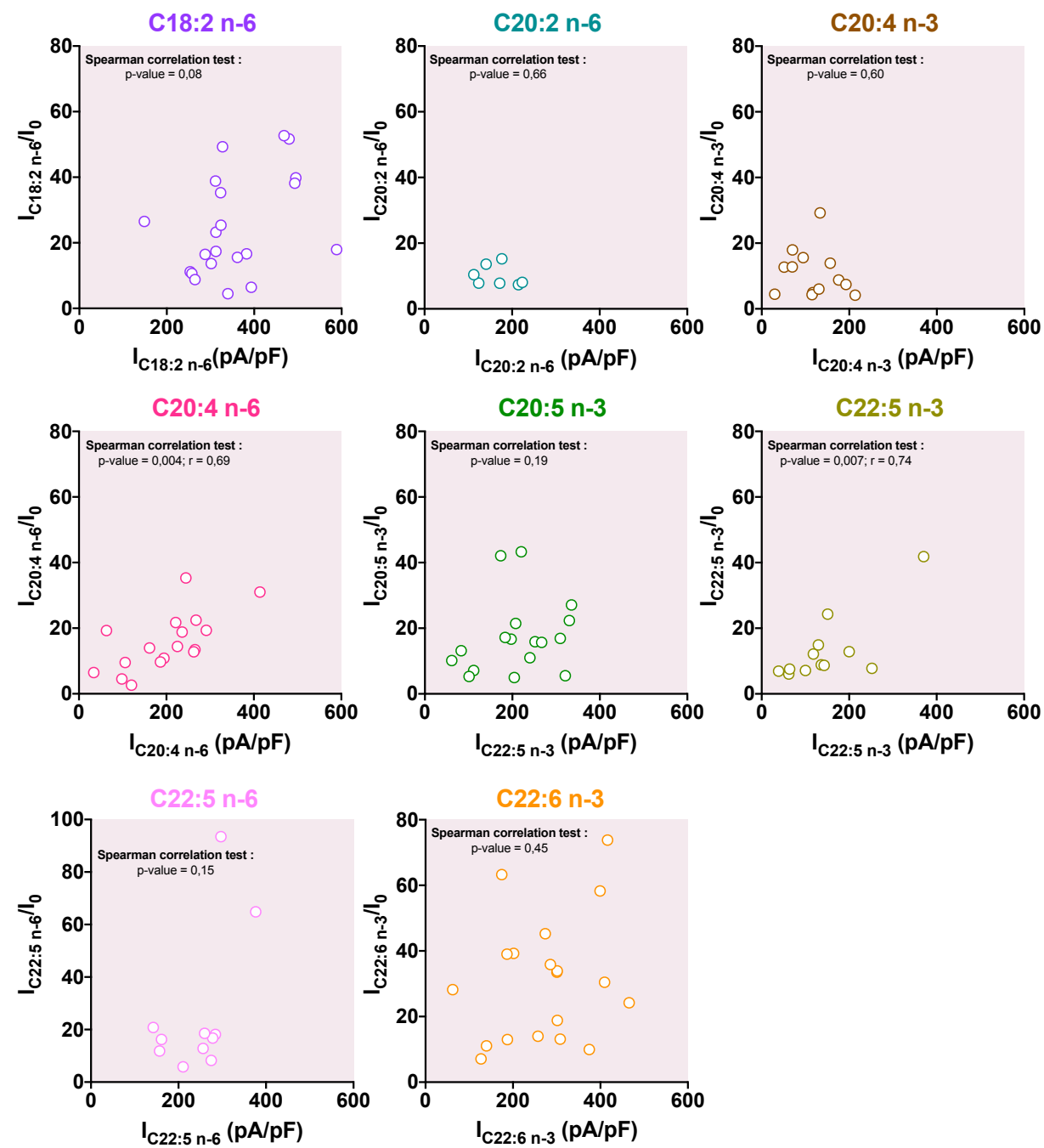

Supplementary Figure 2

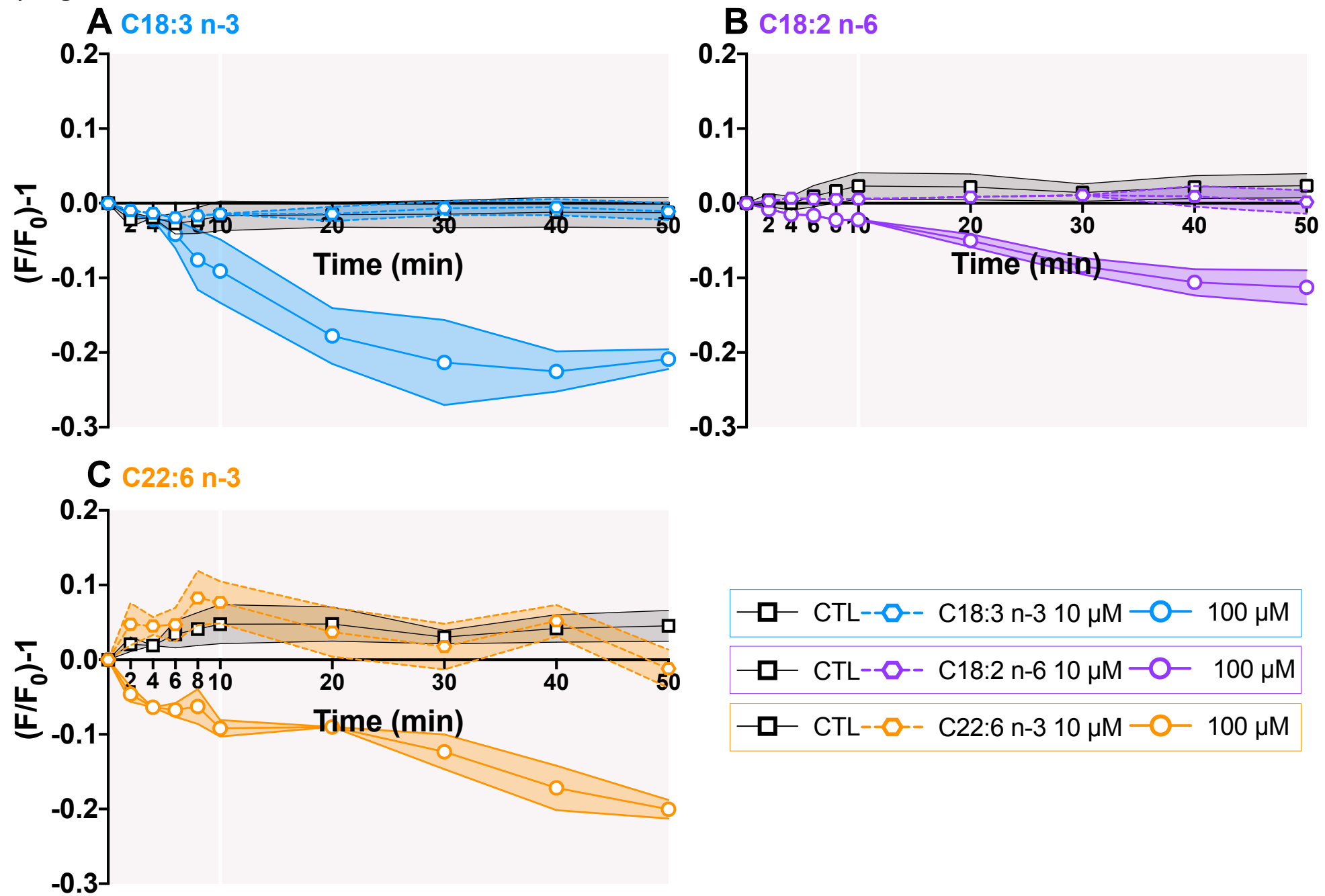

Supplement: Supplementary file 1 — Supplementary Information. [file 41598_2024_66192_MOESM1_ESM.pdf]
